# Long Term Continuous Positive Airway Pressure and Non-invasive Ventilation in Obstructive Sleep Apnea in Children With Obesity and Down Syndrome

**DOI:** 10.3389/fped.2020.00534

**Published:** 2020-08-28

**Authors:** Stijn Verhulst

**Affiliations:** ^1^Lab of Experimental Medicine and Pediatrics, University of Antwerp, Antwerp, Belgium; ^2^Department of Pediatrics, Antwerp University Hospital, Edegem, Belgium

**Keywords:** obstructive sleep apnea, obesity, down syndrome, continuous positive airway pressure, non-invasive ventilation

## Abstract

This review will focus on non-invasive ventilation (NIV) and continuous positive airway pressure (CPAP) therapy in children with obstructive sleep apnea (OSA) due to obesity and underlying syndromes. These children have a high prevalence of OSA and residual OSA after adenotonsillectomy. Therefore, a high proportion of these children are treated with CPAP or NIV. This review will focus on treatment selection tools and will subsequently cover specific issues on CPAP treatment in obese and syndromic children with a major focus on Down syndrome.

## Introduction

Obstructive sleep apnea syndrome (OSAS) is a manifestation of sleep-disordered breathing (SDB) in children. OSAS is characterized by prolonged episodes of increased upper airway (UA) resistance and respiratory effort with partial (obstructive hypopnea) or complete (obstructive apnea) UA obstruction during sleep. The syndrome is often associated with snoring, intermittent hypoxia, hypercarbia, and/or sleep disruption. Additionally, OSAS is associated with a number of significant complications such as daytime neurobehavioral problems, learning deficits, growth retardation, and cardiovascular complications and it should therefore be correctly treated (1, 2).

Structural narrowing of the UA in combination with inadequate compensation for a decrease in UA neuromuscular tone is an important factor in the pathogenesis of OSAS (3, 4). Adenotonsillar hypertrophy is the most important predisposing factor for UA narrowing in otherwise healthy children. However, many other causes of craniofacial defects may coexist such as maxillary and mandibular deficiency, tongue, and soft palate enlargement, and inferior displacement of the hyoid bone (3). Additionally, the pathogenesis of UA narrowing is more complex in certain subgroups such as children with obesity, craniofacial malformation, Down syndrome or neuromuscular disorders. The complexity of the pathogenesis of OSAS in these children is illustrated by a high incidence of residual OSAS after adenotonsillectomy (AT) and by a frequent need for additional treatment. For instance, residual OSAS after AT is reported in 54–88% of obese children compared to 15–26% in non-obese children (5–7). Lumeng et al. (8) also demonstrated that the prevalence of OSAS in these subgroups is markedly increased compared to the prevalence of 1–4% in the general population (8). Obese children have a prevalence ranging from 13 to 59% (9), children suffering from Down syndrome 30–100% (10–14), achondroplasia 54% (15, 16), craniofacial syndromes such as Pierre-Robin sequence 85% (17) and non-syndromic cleft palate 8, 5% (18).

In view of the high prevalence of OSAS and residual OSAS after adenotonsillectomy, a high proportion of these children are treated with continuous positive airway pressure (CPAP) or non-invasive ventilation (NIV). CPAP or NIV is not a first line treatment in these children. In general, it is reserved for children with residual OSA after adenotonsillectomy or other upper airway surgery or in those children who are not surgical candidates. In our opinion, the setting for upper airway surgery should also be set correctly as obesity or Down syndrome are also associated with post-operative complications (1). Therefore, it is critical to identify the anatomical site(s) responsible for obstruction in the upper airway and to couple these findings with the most appropriate treatment. Treatment can consist of (a combination of) weight loss, anti-inflammatory medication, orthodontics, (adeno) tonsillectomy, supraglottoplasty, lingual tonsillectomy, other upper airway surgery or CPAP/NIV most often in the context of multilevel obstruction. This review will briefly focus on these treatment selection tools and will subsequently cover specific issues on positive airway pressure (PAP) treatment in obese and syndromic children with a major focus on Down syndrome.

## Treatment Selection: How to Select Between Surgery vs. NIV?

The European Respiratory Society published two consensus statements from a multidisciplinary expert group describing a diagnostic and treatment algorithm for OSAS in infants and children (1, 2). Especially in infants where adenotonsillar hypertrophy is not yet the predominant cause of UA obstruction, UA endoscopy and imaging are routinely used to identify the anatomical site(s) of UA obstruction. However, it has to be noted that only a limited number of studies with a small sample size evaluated the diagnostic value of endoscopy in infants. Laryngomalacia is a frequent abnormality detected by endoscopy in children with Down syndrome and upper airway obstruction (19). Bravo et al. (20) and Cheng et al. (21) performed endoscopic evaluation in a total of 58 young children (1 month-4 years old) with Pierre Robin sequence. The degree of upper airway obstruction was assessed at the velopharyngeal, oropharyngeal, and tongue base level. Presence of moderate or severe obstruction was 87% sensitive and 100% specific in predicting an obstructive respiratory disturbance index >5 episodes/h (20). Sher et al. (22, 23) described 4 types of pharyngeal airway obstruction in infants and children with various craniofacial abnormalities including craniosynostosis and Pierre Robin sequence: posterior movement of the tongue toward the posterior pharyngeal wall (type I); compression of the soft palate on the posterior pharyngeal wall by the tongue (type II); collapse of the lateral pharyngeal walls (type III); circular constriction of the pharynx (type IV).

In older children, drug induced sleep endoscopy (DISE) and/or imaging are mainly used in the setting of residual OSAS post-adenotonsillectomy. DISE in children with persistent SDB may demonstrate laryngomalacia, adenoidal tissue regrowth, tongue base obstruction, and pharyngeal collapse (16, 24–26). MRI of the upper airway may reveal residual adenoid tissue in obese children with persistent OSAS following adenotonsillectomy (27). Regrown adenoidal tissue, glossoptosis, hypopharyngeal collapse, soft palate collapse, and hypertrophic lingual tonsil are abnormalities that may be identified by cine MRI in children with Down syndrome and persistent SDB after adenotonsillectomy (28, 29).

Although there is a lack of clinical trials concerning this topic, all children with moderate-to-severe OSAS undergo DISE in our center to guide further treatment. This certainly provides more insight into the pattern of UA obstruction in the individual patient. For instance, a study from our group in surgically naïve children with Down syndrome found that the majority of these subjects presented with obstruction at the levels of the adenoids and tonsils. However, 85% of subjects also presented with multilevel collapse which most likely explains the high percentage of residual OSAS after adenotonsillectomy in this cohort (52%) (30).

**Figure 1:**
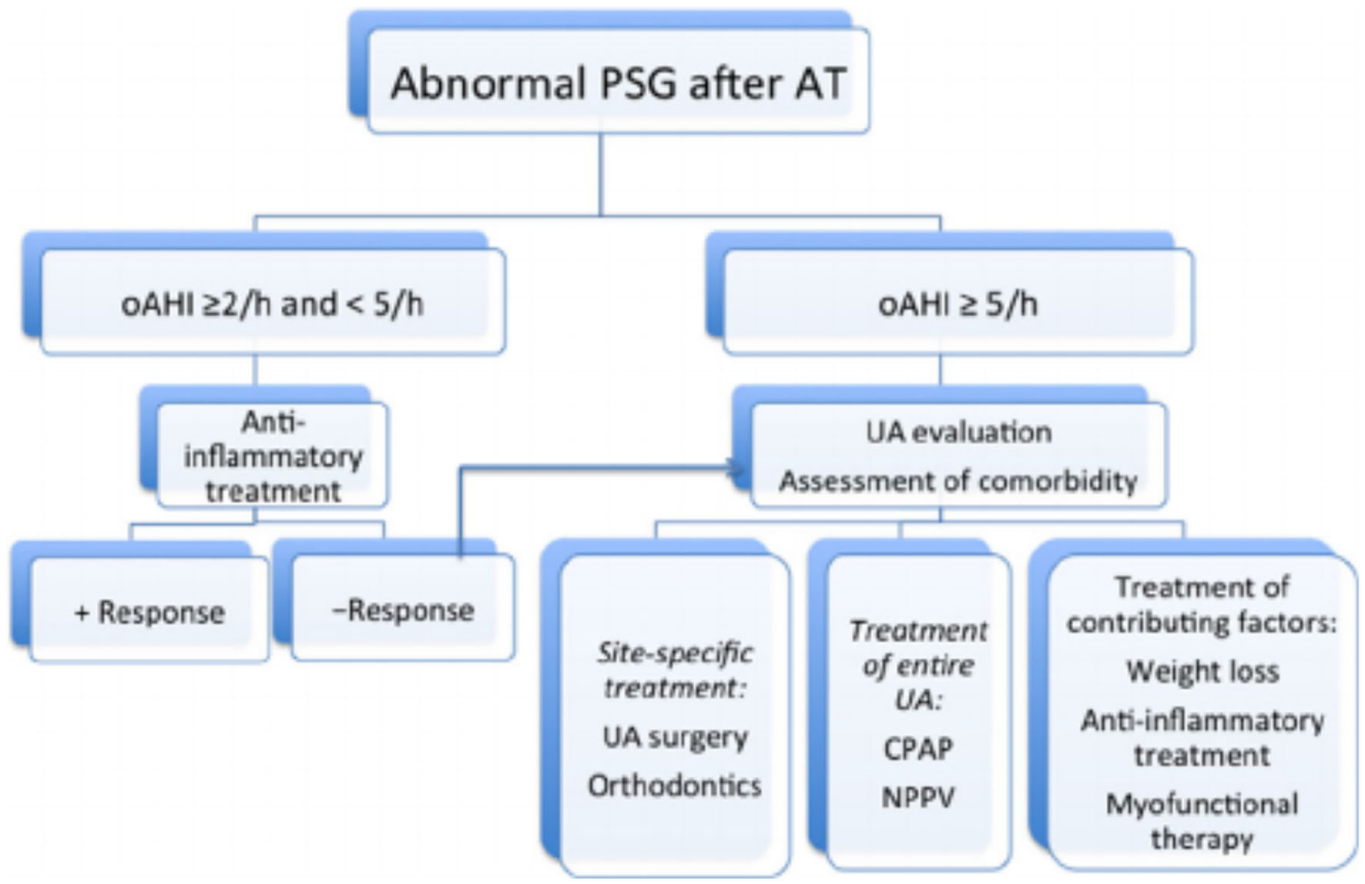
Proposed algorithm for the management of persistent OSA post-adenotonsillectomy (31).

In summary, obesity and the presence of craniofacial malformations or syndromic conditions are major risk factors for residual OSAS. Upper airway evaluation by means of DISE and cine MRI may identify lingual tonsillar hypertrophy and laryngomalacia as the most common anatomical correlates for residual disease. These methods may guide the clinicians to specific surgical interventions or non-surgical treatment modalities such as weight loss, orthodontic treatment, medical treatment and myofunctional therapy and toward CPAP or NIV treatment. An overview of this approach is presented in Figure 1 (31).

## CPAP in Obese Children

Concerning sleep apnea in the context of pediatric obesity, it remains crucial that the child is followed up in a multidisciplinary obesity treatment program. It is important to note that limited data from cohort studies generated from residential treatment centers or reports on bariatric surgery have shown that weight loss is beneficial for OSA (32–34). However, the effects of weight loss on an outpatient basis (35) and in young obese children remain little studied. Furthermore, adenotonsillectomy as a treatment for OSA in obese children is frequently associated with weight gain and treatment failure (36). In summary, it is crucial to emphasize the importance of weight management in the context of the obese child with OSA. However, weight loss takes time and compliance can be challenging and therefore these children are often referred for CPAP therapy in the context of moderate-to-severe OSA. There is limited data on the prevalence obese subjects entering an obesity clinic and needing CPAP therapy. A recent study from Spain in 113 obese children found an OSA prevalence of 55%. Out of the 62 subjects with OSA, 10 were started on CPAP corresponding to an incidence of 16%. Indication for CPAP was defined as moderate-to-severe OSA defined as ASI > 10 without endoscopic evidence of adenotonsillar hypertrophy (37).

Adherence to treatment is a general issue in obese patients and this seems also true for obese children and adolescents requiring CPAP therapy. Marcus et al. (38) compared CPAP adherence between children on bilevel positive airway pressure with pressure release technology (Bi-Flex) and a group on standard CPAP therapy. Fifty six children were included, and the vast majority of these patients were obese. Most subjects attempted to use their ventilation on most nights, with the devices being turned on more than two-thirds of the nights during the first month of therapy. However, the average nightly use varied widely between subjects, ranging from 1 to 536 min/night for the first month and decreased during the following months (38). Another study on adherence by the same group investigated predictors of adherence in a similar cohort. Obesity was not an independent predictor of adherence. Lower maternal education was the strongest predictor of poor adherence, with older, typically developing youth, and African American youth less adherent to CPAP therapy as well. Lower levels of social support were also associated with poor adherence (39). An Australian cohort showed better adherence rates. However, this study noted that 50% of the 35 cases who did not continue in the current study for CPAP were overweight or obese and their failure to comply with therapy commenced very early in the process (i.e., before therapy initiation) (40). Pury et al. (41) studied a cohort of 56 children with a mean age of 13 years and 65% of the studied subjects were obese. CPAP use was the highest at week 1 (used 79% percent of nights), then declined over time: 65% of nights at month 1 and 57% of nights at 3 months, with wide individual variation. Average nightly PAP use also declined over time: from 3.5 ± 2.7 h at week 1 to 2.8 ± 2.4 h at month 3. CPAP was use was better if another family member was also on CPAP, obesity did not affect CPAP adherence (41). Amaddeo et al. (42) studied out-patient initiation of CPAP therapy in children and showed a general high level of adherence in their cohort of 31 children (3 were obese).

Limited research has been published on the effects of CPAP therapy on metabolic and cardiovascular comorbidities of pediatric obesity. Sundaram et al. (43) studied the effects of CPAP therapy in subjects with non-alcoholic fatty liver disease. Nine patients were treated with CPAP for ~3 months with relatively good adherence (73% adherence of total days prescribed and a mean usage per day of 296 ± 126 min). With CPAP treatment, participants had an increased duration of sleep (total sleep time), and repeat polysomnography demonstrated improvement in OSA severity. Their results showed that CPAP improved the severity of liver injury and also selected markers of the metabolic syndrome and reduced oxidative stress. This effect was independent from BMI which even increased during the course of the study (43). This last finding is intriguing and clinically relevant suggesting increased energy expenditure during sleep (32). Alonso-Alvarez et al. (37) studied the effects of different OSA treatments on several markers of the metabolic syndrome. Only a limited number of patients on CPAP were included. This might explain why no significant effects of CPAP directly on metabolic markers were observed (37). Another small-scale study in 11 obese children on CPAP therapy only found a change in leptin levels on CPAP treatment (44).

In summary, we can conclude that OSAS in pediatric obesity is highly prevalent. The pathophysiology is multifactorial, but these children need to be included in a multidisciplinary weight management program in context of the several other obesity-related complications. Weight loss can improve OSAS, but CPAP is indicated in children with moderate-to-severe OSAS in whom surgery is not indicated. Adherence can be an issue in these subjects and these subjects need to be followed closely. The effects of CPAP on cardiovascular and metabolic complications in obese children and adolescents require more study.

## CPAP in Children With Down Syndrome

Our group has published on the incidence of CPAP therapy in children with Doqn syndrome. Maris et al. (30) studied DISE-directed therapy in 41 surgically naïve children with Down syndrome and OSA. Seven percentage of of patients were directly referred to CPAP therapy. Twenty five children underwent (adeno) tonsillectomy but with a high percentage (~50%) of persistent OSA. The majority of these patients had multilevel collapse on DISE. One of these patients was also referred for CPAP therapy (30).

Published research regarding CPAP in Down syndrome is somewhat limited. Trucco et al. (45) described 39 patients (out of a total group of 60) with different kinds of respiratory support: 14 patients on supplemental oxygen, 18 on CPAP, and 7 on bilevel NIV. Median age at initiation of respiratory support was 2.4 years old (interquartile range 0.7–6). Twelve children out of 60 were referred for a sleep study because of OSAS symptoms persisting after adeno-tonsillectomy, while 48 did not receive any surgery at the time of the sleep study. All of the 12 referred post-adenotonsillectomy patients had evidence of significant OSAS requiring ventilatory support. Six started CPAP and three bilevel NIV while two were started on oxygen for lack of tolerance to positive pressure support and one child could not tolerate even nasal cannula with additional oxygen. Out of the 48 surgical naïve patients, 22 out of the 23 children diagnosed with sleep-disordered breathing were started on respiratory support first-line: 10 commenced CPAP, 2 bilevel NIV, 10 required overnight O2 for low overnight saturations or central apneas, whereas 11 patients were referred for adenotonsillectomy after which 6 went on to require initiation of positive pressure therapy for evidence of residual OSAS. After a median of 4 months after initiation of respiratory support, 22 out of 39 (56%) were considered as regular users. Oxygen was reported to be tolerated by 9 out of 14 subjects (64%), 9 out of 18 patients (50%), and 4 out of 7 (57%) had satisfactory adherence to CPAP and bilevel NIV, respectively. The mean CPAP use was 5 h, and median bilevel NIV use was 8 h. Adherence at the latest evaluation, after ~2 years, was reported as good in 6 out of 9 (67%) patients on O2, in 7 out of 18 (39%) patients on CPAP, and in 4 out of 6 (67%) on bilevel NIV (45). Dudoignon et al. (46) described 19 patients on CPAP or NIV therapy. Patients on CPAP or NIV therapy had more severe sleep apnea compared to patients who did not need respiratory support. The mean age at CPAP/NIV initiation was 7 ± 7 years with a wide range (0.4–23 years). Mean duration of treatment at the time of the study was ~2 years. CPAP/NIV adherence was available only in 11 patients, mainly because of a too young age in 4 patients which did not allow an accurate interpretation of actual ventilator use. Adherence was good with an average use per night of almost 9 h and 9/11 patients using CPAP/NIV > 4 h/night. Three patients could be successfully weaned from CPAP/NIV. Finally, no complications were observed with CPAP/NIV (46). Sudarsan et al. (47) compared adenotonsillectomy vs. CPAP in Down syndrome patients with OSA. In the CPAP group, 36 subjects completed the study. Five subjects had persistent OSA (defined as AHI > 1 corresponding to a failure rate of 14%). Children receiving CPAP or adenotonsillectomy had similar improvements in symptoms, quality of life and AHI (47).

Along these lines, adherence issues are frequently encountered. Fortunately, adherence tends to be a greater issue when beginning therapy, and consistent usage can often be achieved with time. For instance, the study by Dudoignon et al. (46) showed that 81% of patients had CPAP usage > 4 h per night 1–3 years after starting therapy. An alternative in patients with CPAP intolerance could be switching to high flow nasal cannula therapy (48) or switching to this interface coupled to a regular ventilator (49). Amaddeo et al. (48) studied HFNC in 8 patients who were intolerant to CPAP (6 patients had Down syndrome). Three out of the 6 patients with Down syndrome were successfully managed with HFNC, the other patients did not tolerate HFNC as well. These three patients all had severe neurocognitive and behavioral impairment (48).

## Conclusion

OSAS in children with obesity and underlying syndromes is highly prevalent. Treatment selection is critical, to limit unsuccessful surgery and in view of the high prevalence of residual OSAS after adenotonsillectomy. Therefore, a high proportion of these children are treated with CPAP or NIV. Overall, PAP therapy has beneficial effects on sleep parameters, daytime symptoms, quality of life, and metabolic parameters. However, these data are generated from a limited number of studies and more studies on the effects of CPAP are certainly warranted. Compliance can be an issue in obese children and patients with Down syndrome for instance, however an intensive initial follow-up can certainly be helpful in these cases. The use of HFNC certainly deserves more study in CPAP intolerant patients.

## Author Contributions

The author confirms being the sole contributor of this work and has approved it for publication.

## Conflict of Interest

The author declares that the research was conducted in the absence of any commercial or financial relationships that could be construed as a potential conflict of interest.
